# Supplementary figures and images for: Silencing of the ARK5 gene reverses the drug resistance of multidrug-resistant SGC7901/DDP gastric cancer cells
Source: PeerJ. 2020 Aug 7;8:e9560. doi: 10.7717/peerj.9560 (PMC7416719; doi:10.7717/peerj.9560)

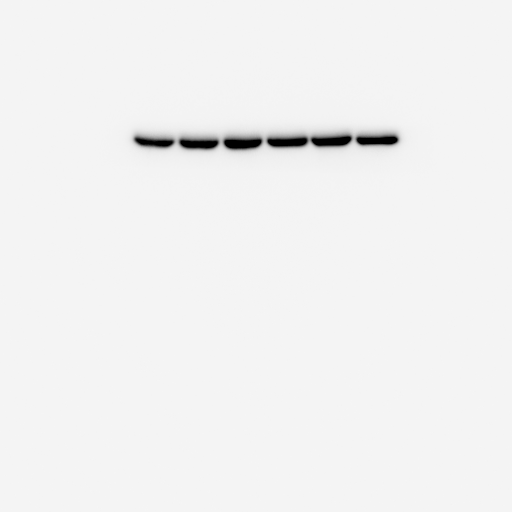

Supplement: Supplemental Information 1 — Including all the images of full-length uncropped blots. [file peerj-08-9560-s001.zip › Original images of electrophoretic gels and blots/Figure 1. actin.tif]

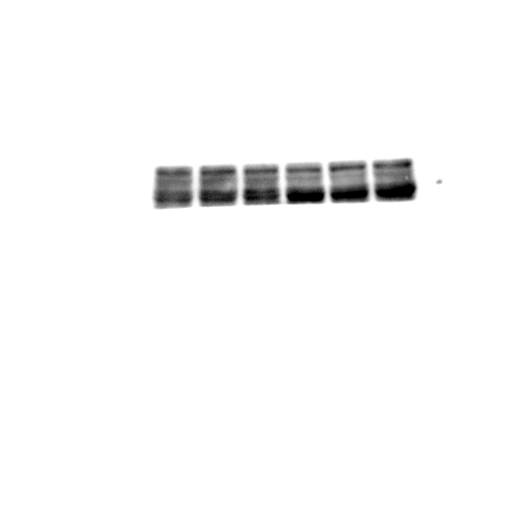

Supplement: Supplemental Information 1 — Including all the images of full-length uncropped blots. [file peerj-08-9560-s001.zip › Original images of electrophoretic gels and blots/Figure 1. ark5.tif]

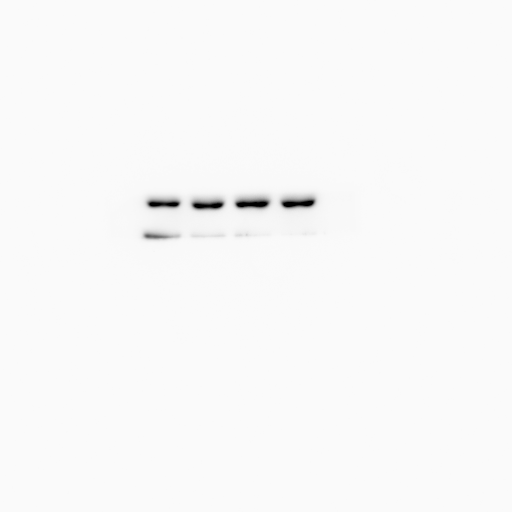

Supplement: Supplemental Information 1 — Including all the images of full-length uncropped blots. [file peerj-08-9560-s001.zip › Original images of electrophoretic gels and blots/Figure 2. actin.tif]

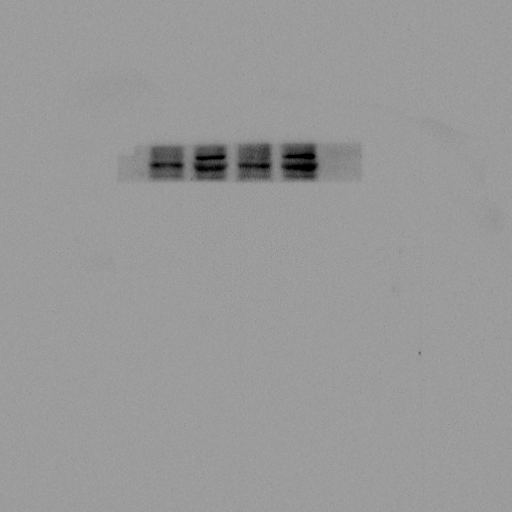

Supplement: Supplemental Information 1 — Including all the images of full-length uncropped blots. [file peerj-08-9560-s001.zip › Original images of electrophoretic gels and blots/Figure 2. ark5.tif]

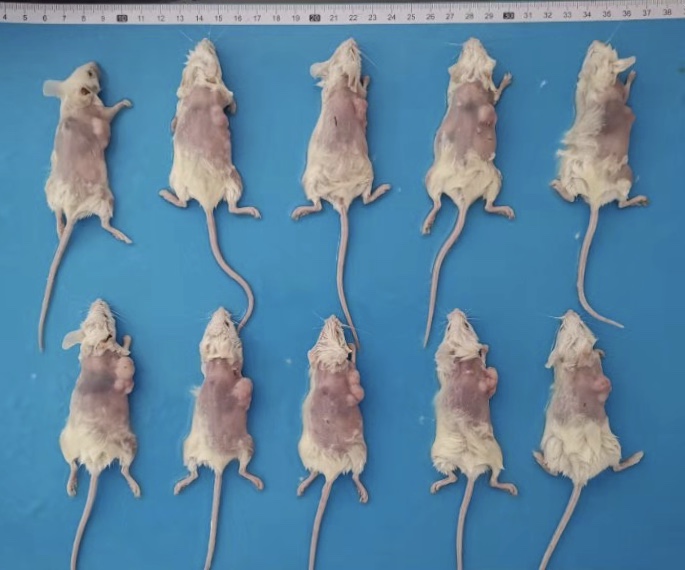

Supplement: Supplemental Information 4 — IIncluding all the raw data and images from the mouse trial. [file peerj-08-9560-s004.zip › Raw data of Figure7/Figure7 A.JPG]

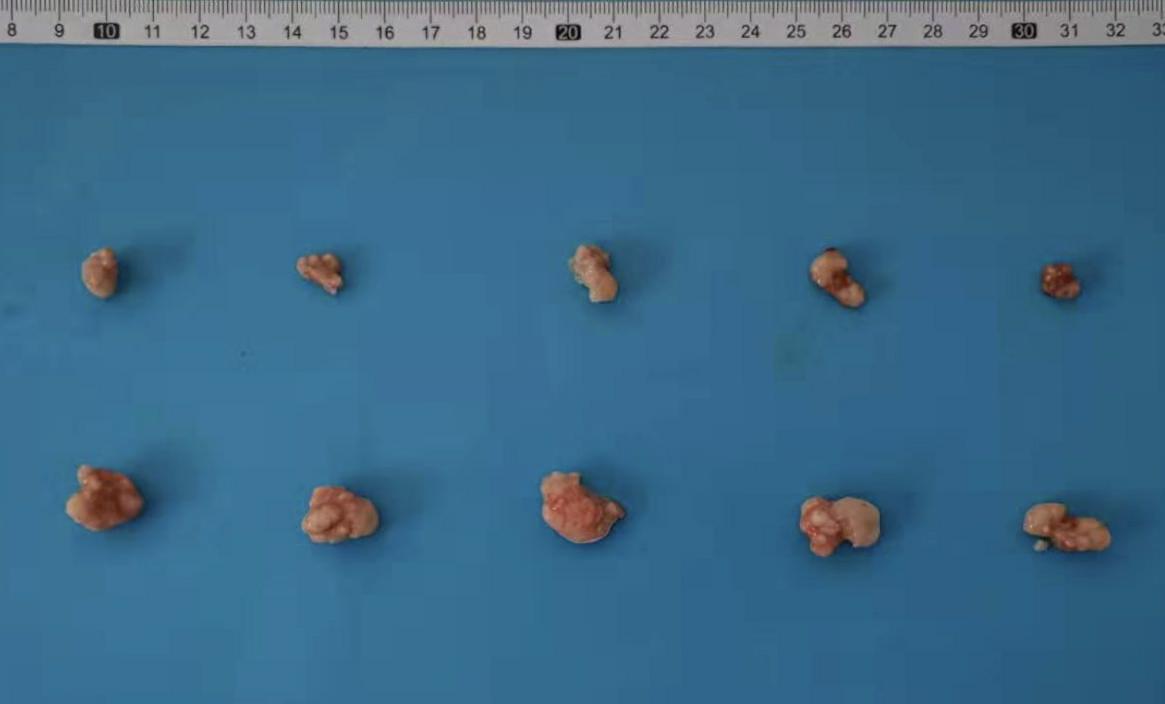

Supplement: Supplemental Information 4 — IIncluding all the raw data and images from the mouse trial. [file peerj-08-9560-s004.zip › Raw data of Figure7/Figure7 C.JPG]
