# Supplementary material for: Silencing of the ARK5 gene reverses the drug resistance of multidrug-resistant SGC7901/DDP gastric cancer cells
Source: PeerJ. 2020 Aug 7;8:e9560. doi: 10.7717/peerj.9560 (PMC7416719; doi:10.7717/peerj.9560)
Supplement: Supplemental Information 5 [file peerj-08-9560-s005.docx]

**References**

1. Frank-Stromborg, M., *The epidemiology and primary prevention of gastric and esophageal cancer. A worldwide perspective.* Cancer Nursing, 1989. **12**(2): p. 53-64.

2. Karimi, P., F. Islami, S. Anandasabapathy, N.D. Freedman, and F. Kamangar, *Gastric Cancer: Descriptive Epidemiology, Risk Factors, Screening, and Prevention.* Cancer Epidemiology Biomarkers & Prevention. **23**(5): p. 700-713.

3. Ferlay, J., I. Soerjomataram, R. Dikshit, S. Eser, C. Mathers, M. Rebelo, D.M. Parkin, D. Forman, and F. Bray, *Cancer incidence and mortality worldwide: Sources, methods and major patterns in GLOBOCAN 2012.* International Journal of Cancer. **136**(5): p. E359-E386.

4. Cornejo, C. and M. Portanova, *[Comparative study of D1 and D2 ganglionic dissection in advanced gastric cancer at Rebagliati Hospital].* Rev Gastroenterol Peru, 2006. **26**(4): p. 351-356.

5. Kang, J.H., S.I. Lee, D.H. Lim, K.-W. Park, S.Y. Oh, H.-C. Kwon, I.G. Hwang, S.-C. Lee, E. Nam, and D.B. Shin, *Salvage Chemotherapy for Pretreated Gastric Cancer: A Randomized Phase III Trial Comparing Chemotherapy Plus Best Supportive Care With Best Supportive Care Alone.* Journal of Clinical Oncology Official Journal of the American Society of Clinical Oncology. **30**(13): p. 1513-1518.

6. Cunningham, S.C., F. Kamangar, P.K. Min, S. Hammoud, R. Haque, A. Maitra, E. Montgomery, R.E. Heitmiller, M.A. Choti, and K.D. Lillemoe, *Survival After Gastric Adenocarcinoma Resection: Eighteen-Year Experience at a Single Institution.* Journal of Gastrointestinal Surgery, 2005. **9**(5): p. 718-725.

7. Isobe, Y., A. Nashimoto, K. Akazawa, I. Oda, K. Hayashi, I. Miyashiro, H. Katai, S. Tsujitani, Y. Kodera, and Y. Seto, *Gastric cancer treatment in Japan: 2008 annual report of the JGCA nationwide registry.* Gastric Cancer, 2011. **14**(4): p. 301-316.

8. Linn, S., A. Honkoop, K. Hoekman, P. van der Valk, H. Pinedo, and G. Giaccone, *p53 and P-glycoprotein are often co-expressed and are associated with poor prognosis in breast cancer.* British Journal of Cancer. **74**(1): p. 63-68.

9. Zhang, X., X. Peng, W. Yu, S. Hou, Y. Zhao, Z. Zhang, X. Huang, and K. Wu, *Alpha-tocopheryl succinate enhances doxorubicin-induced apoptosis in human gastric cancer cells via promotion of doxorubicin influx and suppression of doxorubicin efflux.* Cancer Letters. **307**(2): p. 0-181.

10. Gottesman, M.M., *Biochemistry of Multidrug Resistance Mediated by the Multidrug Transporter.* Annual Review of Biochemistry, 1993. **62**(1): p. 385-427.

11. Chabner, B.A. and A. Fojo, *Multidrug Resistance: P-glycoprotein and Its Allies--The Elusive Foes.* Journal of the National Cancer Institute. **81**(12): p. 910-913.

12. Biedler, J.L. and B.A. Spengler, *Reverse transformation of multidrug-resistant Cells.* Cancer & Metastasis Reviews, 1994. **13**(2): p. 191-207.

13. C, C.-C., O.B. JP, B. J, C. D, B. JR, and M. MR, *Expression of the multidrug resistance gene product (P-glycoprotein) in human normal and tumor tissues.* The journal of histochemistry and cytochemistry : official journal of the Histochemistry Society, 1990. **38**(9): p. 1277-87.

14. Wu, C.P. and S. V. Ambudkar, *The pharmacological impact of ATP-binding cassette drug transporters on vemurafenib-based therapy.* Acta Pharmaceutica Sinica B. **4**(2): p. 105-111.

15. X, X. and L. XJ, *Overcoming drug efflux-based multidrug resistance in cancer with nanotechnology.* Chinese journal of cancer, 2012. **31**(2): p. 100-9.

16. Goldstein, L.J., H. Galski, A. Fojo, M. Willingham, S.-L. Lai, A. Gazdar, R. Pirker, A. Green, W. Crist, and G.M. Brodeur, *Expression of Multidrug Resistance Gene in Human Cancers.* J Natl Cancer Inst. **81**(2): p. 116-124.

17. D, Z. and F. D, *New insights into the mechanisms of gastric cancer multidrug resistance and future perspectives.* Future oncology (London, England), 2010. **6**(4): p. 527-37.

18. Gürel, S., Ö. Yerci, G. Filiz, E. Dolar, and F. Memik, *High Expression of Multidrug Resistance-1 (MDR-1) and its Relationship with Multiple Prognostic Factors in Gastric Carcinomas in Patients in Turkey.* Journal of International Medical Research, 1999. **27**(2): p. 79-84.

19. JH, C., L. HY, J. HJ, K. HS, Y. JW, K. HC, C. YK, K. MW, and L. KB, *Expression of multidrug resistance-associated protein1,P-glycoprotein, and thymidylate synthase in gastric cancer patients treated with 5-fluorouracil and doxorubicin-based adjuvant chemotherapy after curative resection.* British journal of cancer, 2002. **86**(10): p. 1578-85.

20. Fan, K.C., D.M. Fan, F.C. Liu, and C.H. Li, *Expression of multidrug resistance-related markers in gastric cancer.* Anticancer Research, 2000. **20**(6C): p. 4809-4814.

21. CC, L., C. JH, T. FJ, H. YM, J. YN, Y. JS, and C. HY, *Metformin triggers the intrinsic apoptotic response in human AGS gastric adenocarcinoma cells by activating AMPK and suppressing mTOR/AKT signaling.* International journal of oncology, 2019. **54**(4): p. 1271-1281.

22. Hardie, D.G., *AMP-activated protein kinase—an energy sensor that regulates all aspects of cell function.* 2011. **25**(18): p. 1895-1908.

23. HW, C., L. YS, N. HY, H. MW, K. HJ, M. SY, J. H, P. JJ, C. TE, C. SE, K. SW, and K. SY, *Knockdown of β-catenin controls both apoptotic and autophagic cell death through LKB1/AMPK signaling in head and neck squamous cell carcinoma cell lines.* Cellular signalling, 2013. **25**(4): p. 839-47.

24. Yu, Z., H. Cheng, H. Zhu, M. Cao, and Y. Li, *Salinomycin enhances doxorubicin sensitivity through reversing the epithelial-mesenchymal transition of cholangiocarcinoma cells by regulating ARK5.* Brazilian Journal of Medical & Biological Research, 2017. **50**(10).

25. Xu, T., J. Zhang, W. Chen, S. Pan, X. Zhi, L. Wen, Y. Zhou, B.W. Chen, J. Qiu, and Y. Zhang, *ARK5 promotes doxorubicin resistance in hepatocellular carcinoma via epithelial–mesenchymal transition.* Cancer Letters: p. S0304383516302610.

26. Liu, J., T. Guoyan, H. He, L. Huan, Z. Peng, and X. Lihua, *Expression level of NUAK1 in human nasopharyngeal carcinoma and its prognostic significance.* European Archives of Oto-Rhino-Laryngology.

27. Suzuki, A., J. Lu, G.I. Kusakai, A. Kishimoto, and H. Esumi, *ARK5 Is a Tumor Invasion-Associated Factor Downstream of Akt Signaling.* Molecular & Cellular Biology, 2004. **24**(8): p. 3526-3535.

28. SA, H., B. J, R. JL, M. KJ, U. L, M. TP, A. DR, and H. DG, *Complexes between the LKB1 tumor suppressor, STRAD alpha/beta and MO25 alpha/beta are upstream kinases in the AMP-activated protein kinase cascade.* Journal of biology, 2003. **2**(4): p. 28.

29. M, M., H. SP, and C. M, *Mammalian TAK1 activates Snf1 protein kinase in yeast and phosphorylates AMP-activated protein kinase in vitro.* The Journal of biological chemistry, 2006. **281**(35): p. 25336-43.

30. *Deregulated MYC expression induces dependence upon AMPK-related kinase 5.* Nature. **483**(7391): p. 608-612.

31. Li, M., C. Zheng, H. Xu, W. He, and W. Li, *Inhibition of AMPK-related kinase 5 (ARK5) enhances cisplatin cytotoxicity in non-small cell lung cancer cells through regulation of epithelial-mesenchymal transition.* American Journal of Translational Research, 2017. **9**(4): p. 1708-1719.

32. Chen, D., G. Liu, N. Xu, X. You, H. Zhou, X. Zhao, and Q. Liu, *Knockdown of ARK5 Expression Suppresses Invasion and Metastasis of Gastric Cancer.* Cellular Physiology & Biochemistry, 2017. **42**(3): p. 1025-1036.

33. Friedman, E.A., *New chemotherapeutic drug sensitivity for colon carcinomas in monolayer culture.* Cancer Research, 1988. **48**(11): p. 3236-3244.

34. V, B., B. S, M. K, Z. R, R. H, and T. R, *Lipodox® (generic doxorubicin hydrochloride liposome injection): in vivo efficacy and bioequivalence versus Caelyx® (doxorubicin hydrochloride liposome injection) in human mammary carcinoma (MX-1) xenograft and syngeneic fibrosarcoma (WEHI 164) mouse models.* BMC cancer, 2017. **17**(1): p. 405.

35. CL, D., X. HY, T. LF, Z. X, L. YJ, Z. MS, C. LM, W. XH, and F. LW, *Tetrandrine achieved plasma concentrations capable of reversing MDR in vitro and had no apparent effect on doxorubicin pharmacokinetics in mice.* Cancer chemotherapy and pharmacology, 2007. **60**(5): p. 741-50.

36. 张慧, *KLF8在缺氧诱导胃癌细胞多药耐药中的作用及机制研究*. 2013, 第四军医大学.

37. Yue, Z., L. Chao, X. Fei, C. Wei, Z. Xiao, X. Feng, X. Bai, and T. Liang, *Salinomycin decreases doxorubicin resistance in hepatocellular carcinoma cells by inhibiting the &#x3B2;-catenin/TCF complex association via FOXO3a activation.* Oncotarget. **6**(12).

38. Wang, X., *Preliminary Study on Preparation and Pharmaceutic Features of Adrimycin-loaded Human Serum Albumin Microspheres.* Journal of Sichuan University, 2004. **35**(1): p. 107.

39. M?hler, M., *New Perspectives in the Treatment of Advanced Gastric Cancer: S-1 as a Novel Oral 5-FU Therapy in Combination with Cisplatin.* Chemotherapy: p. 62-70.

40. Von Kalle, C., I. Fidler, A.V. Deimling, R. Giavazzi, S. Naito, and I.J. Fidler, *Growth and metastasis of tumor cells isolated from a human renal cell carcinoma implanted into different organs of nude mice.* 1986. **46**(8): p. 4109.

41. C, N., P. A, S. A, and L. H, *Modulation of the classical multidrug resistance (MDR) phenotype by RNA interference (RNAi).* FEBS letters, 2003. **545**(null): p. 144-50.

42. EA, A., F. MF, S.E.S. V, M.N. MC, G.J. JL, A. DV, O. LM, B. ME, T. MS, A.V. S, P.d.A. V, D. AL, A.L.d.M. C, and C. LT, *MRP1 expression in CTCs confers resistance to irinotecan-based chemotherapy in metastatic colorectal cancer.* International journal of cancer, 2016. **139**(4): p. 890-8.

43. V, M. and P. L, *Involvement of p53 in the repair of DNA double strand breaks: multifaceted Roles of p53 in homologous recombination repair (HRR) and non-homologous end joining (NHEJ).* Sub-cellular biochemistry, 2014. **85**(undefined): p. 321-36.

44. N, M., S. M, M. A, S. Y, and S. S, *The evaluation of gastric cancer sensitivity to 5-FU/CDDP in terms of induction of apoptosis: time- and p53 expression-dependency of anti-cancer drugs.* Oncology reports, 2005. **14**(3): p. 609-15.

45. S, C., G. F, D.F. E, S. MP, L. M, C. A, M. P, and C. G, *Expression of p53 protein and resistance to preoperative chemotherapy in locally advanced gastric carcinoma.* Cancer, 1998. **83**(9): p. 1917-22.
